# Phytochemical profiling of *Piper crocatum* and its antifungal activity as Lanosterol 14 alpha demethylase CYP51 inhibitor: a review

**DOI:** 10.12688/f1000research.125645.1

**Published:** 2022-09-28

**Authors:** Tessa Siswina, Mia Miranti Rustama, Dadan Sumiarsa, Dikdik Kurnia

**Affiliations:** 1 Chemistry, Padjadjaran University, Sumedang, Jawa Barat, 45363, Indonesia; 2 Midwifery, Poltekkes Kemenkes Pontianak, Pontianak, Kalimantan Barat, 78124, Indonesia; 3 Biology, Padjadjaran University, Sumedang, Jawa Barat, 45363, Indonesia

**Keywords:** Piper crocatum, antifungal, phytochemical profiling, lanosterol 14 alpha demethylase, CYP51

## Abstract

Mycoses or fungal infections are a general health problem that often occurs in healthy and immunocompromised people in the community. The development of resistant strains in *Fungi *and the incidence of azole antibiotic resistance in the Asia Pacific which reached 83% become a critical problem nowadays. To control fungal infections, substances and extracts isolated from natural resources, especially in the form of plants as the main sources of drug molecules today, are needed. Especially from *Piperaceae*, which have long been used in India, China, and Korea to treat human ailments in traditional medicine. The purpose of this review was to describe antifungal activity from *Piper crocatum* and its phytochemical profiling against lanosterol 14 alpha demethylase CYP51. The methods used search databases from Google Scholar to find the appropriate databases using Preferred Reporting Items for Systematic Reviews and Meta-analyses (PRISMA) flow diagram as a clinical information retrieval method. From 1,150,000 results search by database, there were 73 selected articles to review. The review shows that *P. crocatum* contains flavonoids, tannins, terpenes, saponins, polyphenols, eugenol, alkaloids, quinones, chavibetol acetate, glycosides, triterpenoids or steroids, hydroxychavikol, phenolics, glucosides, isoprenoids, and non-protein amino acids. Its antifungal mechanisms in fungal cells occur due to ergosterol especially lanosterol 14 alpha demethylase CYP51 inhibition as a result of 5,6 desaturase (ERG3) downregulation. *P. crocatum* has an antifungal activity by its phytochemical profiling that act against fungi by inhibiting the fungal cytochrome P 450 pathway, make damaging cell membranes, fungal growth inhibition, morphological changes, and fungal cell lysis.

## Introduction

Mycoses or fungal infections are a general health problem that often occurs in healthy and immunocompromised people in the community ( Ramírez *et al.* 2013). Fungi are divided into four classes: yeasts, filamentous, dimorphic, and dermatophytes; generally, and ubiquitous in the environment, and become pathogenic when immune cells decrease ( Howard *et al.* 2020). Fungal cells have essentially dynamic structure walls for morphogenesis, pathogenesis, and cell viability and act as a dynamic organelle, and need one-fifth of the yeast genome for cell wall biosynthesis ( Gow *et al.* 2017). The development of resistant strain in fungi and the incidence of azole antibiotic resistance in the Asia Pacific which reached of 83% become a critical problem nowadays ( Canuto and Rodero 2002; Jianhua and Hai 2009; Whaley and Rogers 2016; Whaley *et al.* 2017).

The azoles are commonly used because cheaper and have a broad spectrum of antimicrobials ( Lewis 2011; Rosam *et al.* 2021). To control fungal infections, substances, and extracts isolated from natural resources, especially in the form of plants as the main sources of drug molecules today, are needed ( Moghadamtousi *et al.* 2014; Balouiri *et al.* 2016). Natural products have limited or no side effects on human and animals antifungal activity ( Tabassum and Vidyasagar 2013). Antifungal mechanisms in fungal cells occur due to ergosterol inhibition as a result of 5,6 desaturase (ERG3) downregulation ( Neelofar *et al.* 2011). Ergosterol at the fungal plasma membrane is the most common sterol and binding at lanosterol 14α demethylase, an ergosterol-specific enzyme that can cause lanosterol demethylation ( Loeffler and Stevens 2003; Ashley *et al.* 2006; Cannon *et al.* 2007; Emami *et al.* 2017).

*Piperaceae* plant extracts have long been used in India, China, and Korea to treat human ailments in traditional medicine ( Jeon *et al.* 2019). The part of the *Piperaceae* family which has large species of up to 1000 is the *Piper* genus ( Durant-Archibold *et al.* 2018). *Piper* can be found in temperate regions with tropical and sub-tropical ( Lima *et al.* 2020). Indonesia is located in the equator, which has a tropical climate with high humidity and has many natural and biological resources ( Puspita *et al.* 2018). The seeds and leaves of *Piper* species are often cultivated and consumed for various diseases treatment such as antifungal, antibacterial, and disinfectant effects ( Astuti *et al.* 2014; Mgbeahuruike *et al.* 2017).

Isolation of several secondary metabolites of *Piper* species shows that therapeutically molecules like lignans, flavones, alkaloids, unsaturated amides, long and short-chain esters, aristolactams, monoterpenes, sesquiterpenes, ketones, aldehydes, arylpropanoids, chalcones, propenylphenols, and amide alkaloids as a typical constituent ( Gutierrez *et al.* 2013). However, no tests were found on specific compounds for antibacterial activity from *Piper* ( Barh *et al.* 2013). Based on the literature, antifungal compounds are classified into flavonoids, amides, acid derivatives, lignans, prenylated benzoic, cyclopentanedione, butenolides, and phenylpropanoids ( Xu and Li 2011). Of the various *Piper* species, the main constituent is amide, which is classified as aristolactams, open-chain alkamides, amides with pyrolidine, 4,5-dioxoaporphines, Piperidine, and Piperidone groups, ceramides, cyclohexanamid, and cyclobutanamide ( Do Nascimento *et al.* 2012). In this review, we summarize the antifungal activity properties, structural studies, and bio-mechanism of *P. crocatum*, which is commonly found worldwide, against lanosterol 14 alpha demethylase CYP51 in fungi. This review is expected to allow to find new alternative antifungal treatment agents from natural resources based on their active chemical compounds and activities to cure fungal infections, thus reducing the extensive and inappropriate use of antibiotics for antifungal treatment.

## Methods

**Figure 1.**
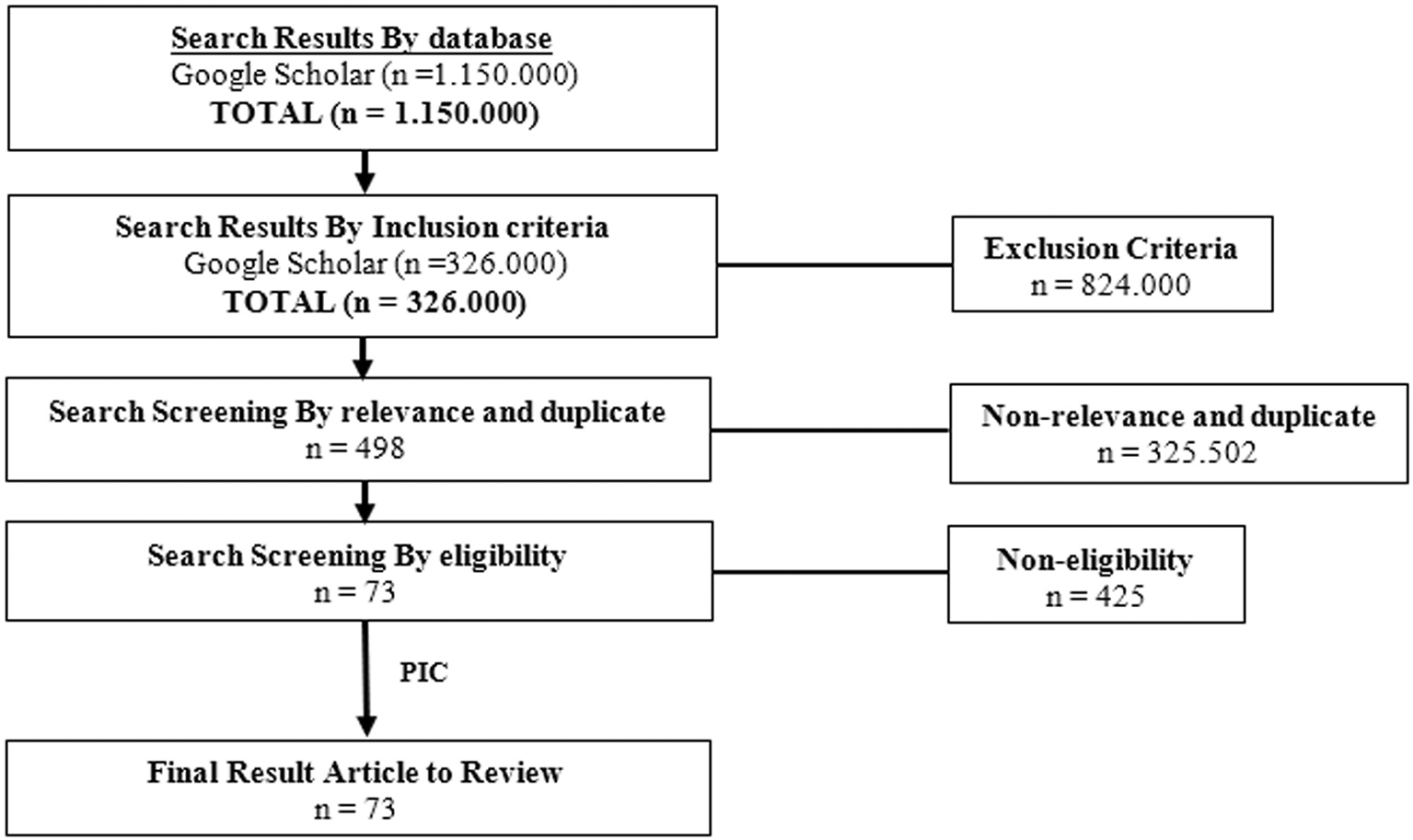
The flowchart of literature reviews of *P. crocatum.*

The author searches databases from Google Scholar to find the appropriate databases using the Preferred Reporting Items for Systematic Reviews and Meta-analyses (PRISMA) Flow Diagram as a clinical information retrieval method. The search screening occurred through four stages. The first stage was screening by the keywords, the second stage was screening by the criteria, the third stage was screening by relevance and duplicates, the fourth stage was screening by eligibility. The keyword of this search was ‘ *Piper’* which yielded 1,150,000 results. The second step was screening by inclusion criteria of the keyword ‘ *Piper’* that reported articles published from 2003 until 2022, which yielded 326,000 results. For the third step, screening by relevance and duplicate with the keyword of this search is ‘ *Piper crocatum’* AND ‘antifungal’, which yielded about 498 results. The final screening results included 73 articles to review. This search was conducted from February to May 2022. The criteria for this research was clinical trials in animal testing and human, books, laboratory tests, case studies, article reviews, systematic reviews, narrative reviews, and meta-analyses. The study was conducted with a true-experimental (Double-Blind RCT), quasi-experimental, study protocol, or pilot study. Articles were published in English. The flow chart of the Literature Review showed in Figure 1.

## Results and discussion

### Ethno-botany and ethno-pharmacology of *P. crocatum*

*P. crocatum*, red betel, a shrub that consists of trunked, 5-10 cm continuous branches, with each segment having roots growing ( Safithri and Fahma 2008). The stems of red betel are round, purplish-green heart-shaped leaves, have no flowers, green with grayish-white tapered ends, special aroma, and very bitter ( Hermiati *et al.* 2013).

### The phytochemical and chemical structures of *P. crocatum*

Isolation of several secondary metabolites of *P. crocatum* contains of flavonoids, tannins, terpenes, saponins, polyphenols, eugenol ( **1**), alkaloids, quinones ( **2**), chavibetol acetate, glycosides ( **3**), triterpenoids ( **4**) or steroids, hydroxychavikol ( **5**), phenolics, glucosides ( **6**), isoprenoids, and non-protein amino acids ( Erviana 2011; Gutierrez *et al.* 2013; Saputra *et al.* 2016; Li *et al.* 2019; Lister *et al.* 2020; Safithri *et al.* 2020).

**Figure 2.**
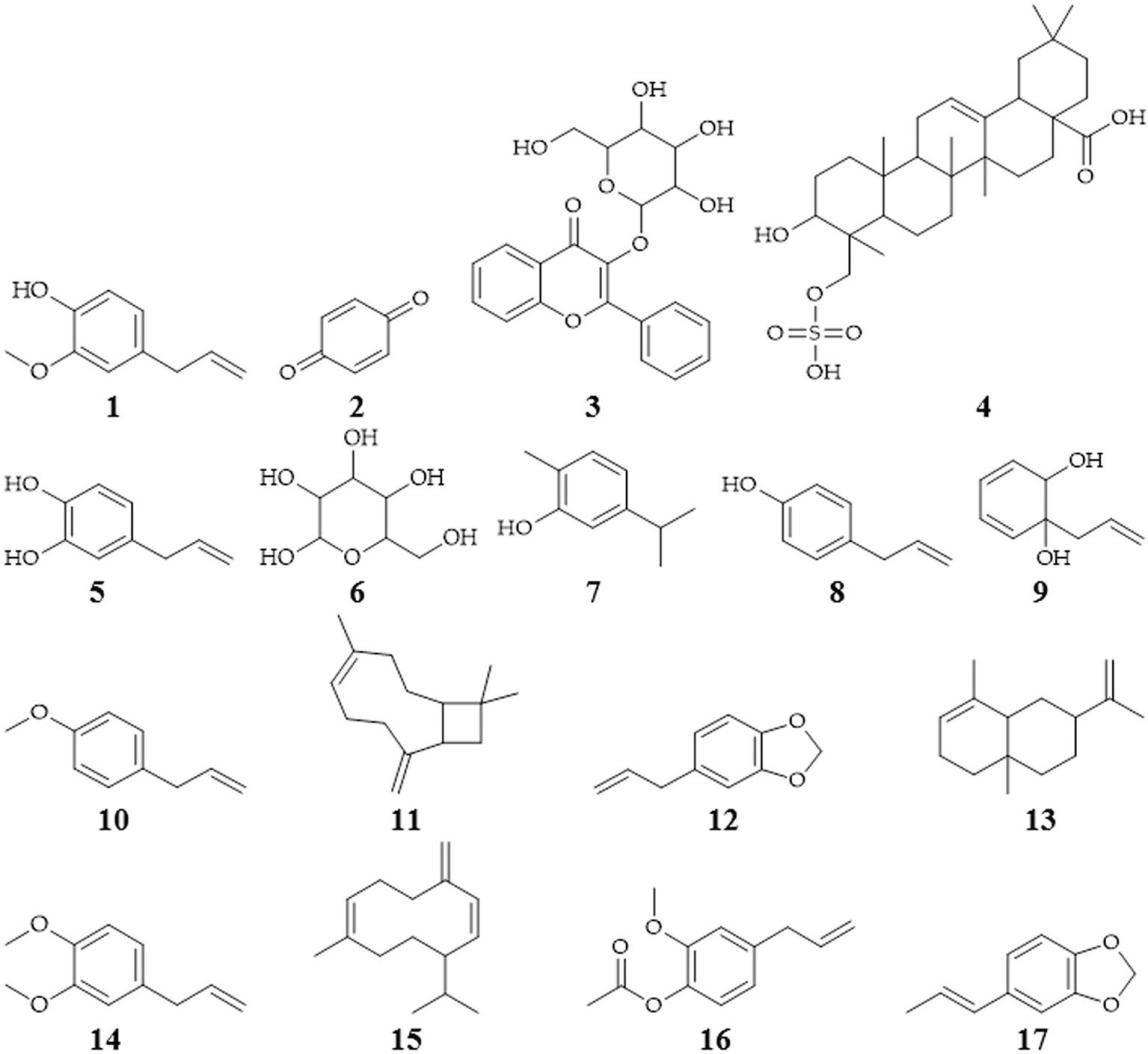
Chemical structures of *P. crocatum* ( Saputra *et al. *2016; Fatmawaty *et al.* 2019; Lister *et al.* 2020).

The bioactive compound of *P. crocatum* as follows Table 1 and Figure 2.

**Table 1.** The bioactive compound of *P. crocatum* ( Suri *et al.* 2021).

Extract type	Chemical compound	Percentage
Betel leaf extract	Alkaloids	
	Carbohydrate	
	Water	
	Tannins	
	Phenol	
	Flavonoids	
	Essential oil	
Betel leaf essential oil	Carvacrol (7)	
	Eugenol ( **1**)	28.44
	Chavicol ( **8**)	
	Allylcatechol ( **9**)	
	Cinema	
	Estragole ( **10**)	
	Caryophyllene ( **11**)	
	Pcymenedaneugenol Metil eter-19	
	Safrole ( **12**)	27.48
	Selinene ( **13**)	7.32
	Methyl eugenol ( **14**)	1.46
	Germacrene D ( **15**)	0.91
	Eugenyl Acetate ( **16**)	1.72
	Isosafrole ( **17**)	1.62

### Test methods and antifungal properties of *P. crocatum*

*P. crocatum* has been tested to *C. albicans* with agar diffusion method ( Kusuma, Manan, *et al.* 2017), MIC determination with microdilution method, and MBC determination by the medium surface of Mueller Hinton Agar, and show that extract of ethanol indicates antifungal activity, MIC value 1.25-2.5% w/v, and MBC value 0.75 min ( Kusuma, Hendriani, *et al.* 2017). The study shows about ten (10) known compounds (2,5-dimethoxy-3-glucopyranosylcinnamic alcohol, cimidahurinin, erigeside II, syringin, β-phenylethyl β-D-glucoside, methyl salicylate 2- *O*-β-D-glucopuranoside, icariside D1, 4-hydroxybenzoic acid β-D-glucosyl ester, benzyl β-D-glucoside, and phenylmethyl 6- *O*-α-L-arabinofuranosyl-β-D-glucopyranoside), and two new phenolic glucosides (Pipercroside A and B) that isolated from MeOH extract of *P. crocatum* elucidated by spectroscopy 1D and 2D NMR, HR-ESI-MS analysis also report erigeside II have the best antifungal activity with IC _50_ value as 58.5 ( Li *et al.* 2019).

Polyphenol inactivates protein and inhibits enzymes on the surface of bacterial cells; flavonoids form complexes that interfere with the function of the bacterial cell wall, inactivating microbial adhesion, enzymes, and cell protein transport by binding to bacterial extracellular proteins through hydrogen and covalent bonds; saponins have hydrophilic molecules and lipid thinning molecules (lipophilic) so that they can make lower cell surface pressure; tannins functions to form complex compounds with enzymes and substrates, thereby disrupting cell membranes, and phenol has hydroxyl and carbonyl groups that can interact with fungal cells through hydrogen bonds, thereby increasing protein coagulation and fungal cell membranes which will cause fungal cells to lyse ( Januarti *et al.* 2019). Inhibition of fungal activity can be done by bother cell membranes, the activity of enzyme and fungi genetic mechanisms ( Ejele *et al.* 2012).

### Antifungal properties and structure

**Figure 3.**
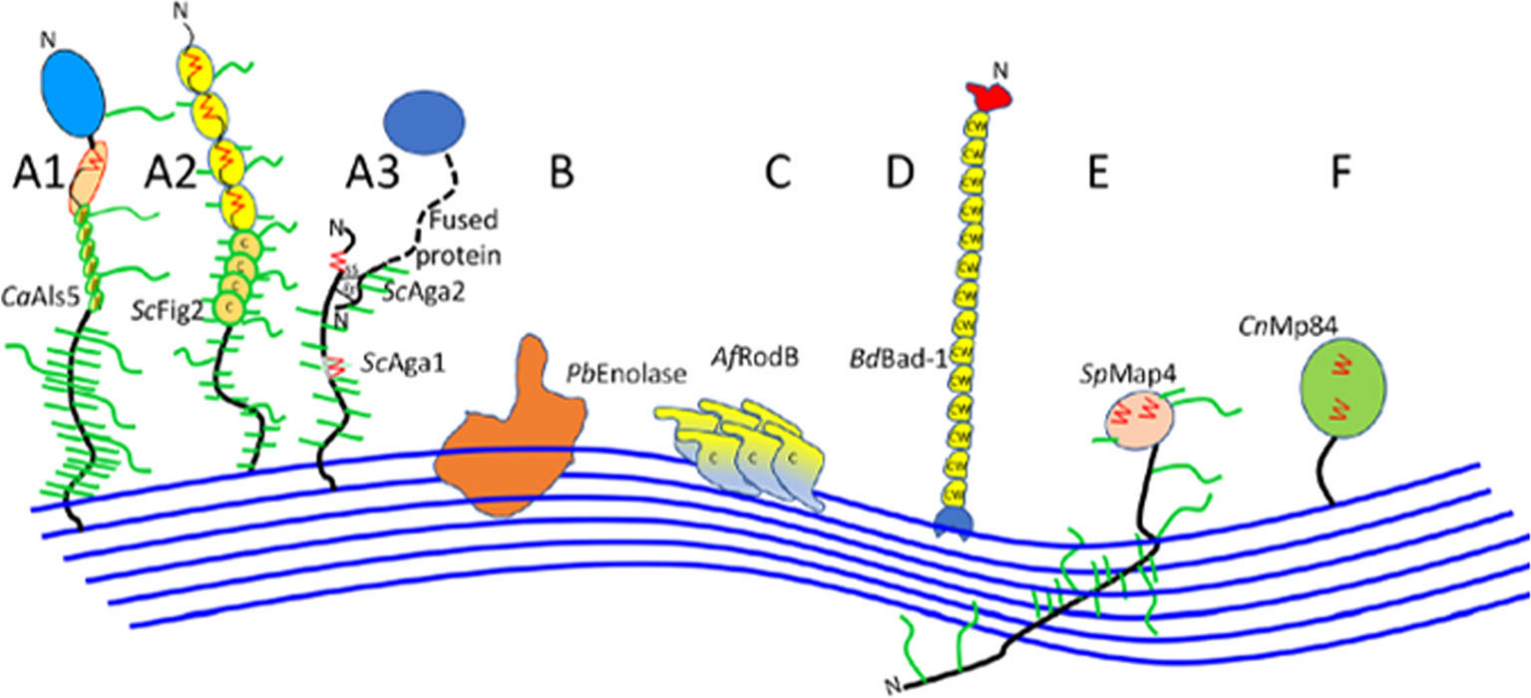
Ilustratiug of cell wal association in fungal adhesins (Ca, *C. albicans*; Sc, *S. cerevisiae*; Pb, *P. braziliensis*; Af, *A. fumigatus*; Bd, *B. dermatitidis*; Sp, *S. pombe*; Cn, *C. neoformans*) ( Lipke 2018).

Fungal cell walls, fungal-specific, serve as a protection from harmful environments and are aggressive because of its toxic and hydrolytic molecules. Ninety percent consist of polysaccharides, with *Saccharomyces* and *Candida* subphylum as the well-characterized fungal adhesins ( Figure 3) ( Latgé 2007; Gow *et al.* 2017). The core of the central fungal cell wall consists of chitin that is linked to branched β-1,3-glucan, combined with galactomannan, galactosamynoglycan, and β-1,3-1,4-glucan in *A. fumigatus* and β-1,6-glucan in *C. albicans* ( Adams 2004; Latgé 2010). Chitin, with a weight of 1-2% from dry cell wall yeast, is the important structure that consists of a homopolymer of β-1,4-linked *N*-acetylglucosamine that is long and linear, while disrupted chitin synthesis will cause the fungal cell wall to be lysis and unstable in osmotic ( Bowman and Free 2006).

**Figure 4.**
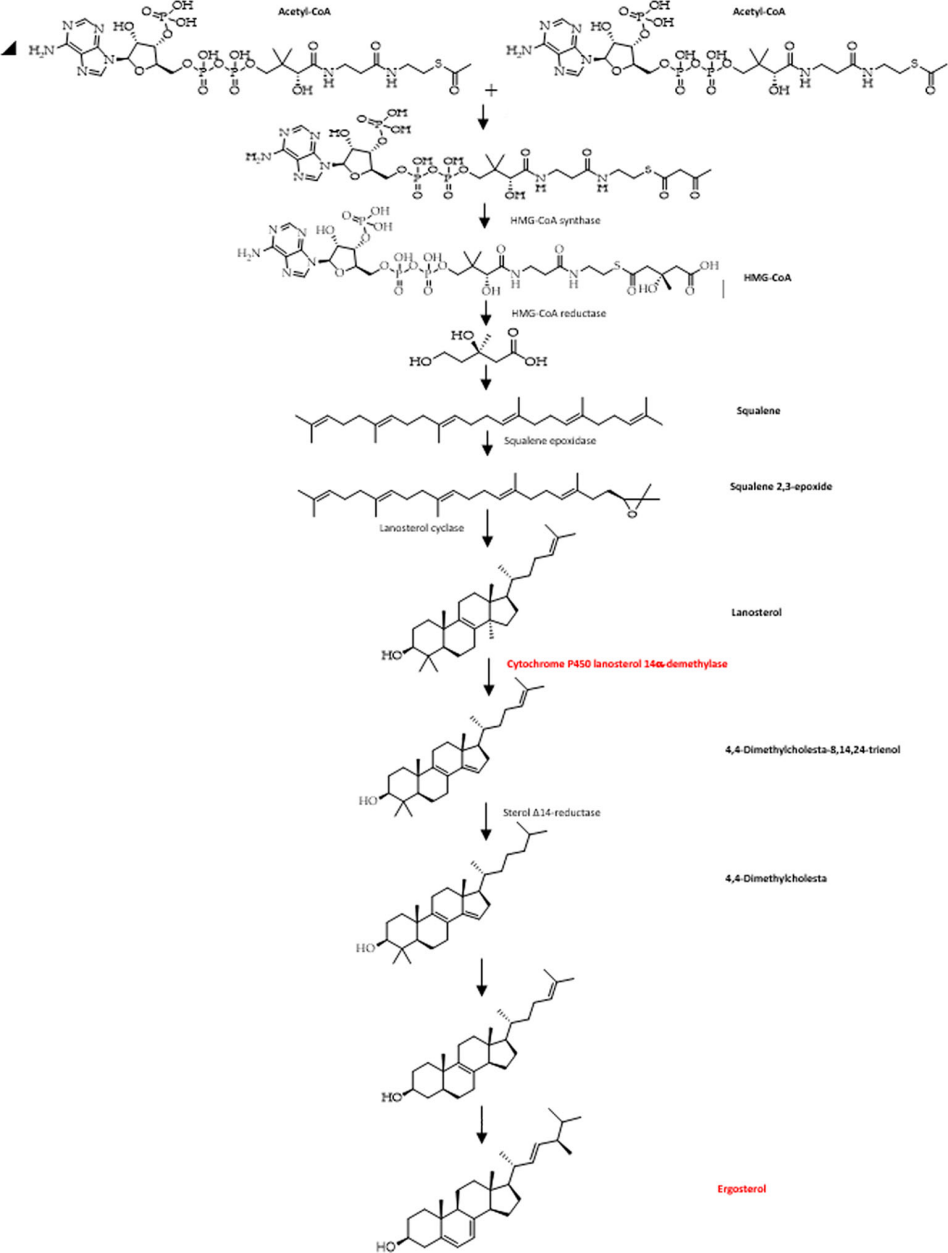
The diagram of ergosterol formation in fungal cells ( Emami *et al.* 2017).

Ergosterol is a key enzyme in fungal-specific sterols, cytochrome P450 enzyme in fungi derived from *S. cerevisiae*, belonging to the CYP51 (lanosterol 14-α-demethylase) family, so with inhibition of biosynthesis in ergosterol, will damage cell membranes ( Arthington-Skaggs *et al.* 1999; Flowers *et al.* 2015). Fungal cell walls that have been damaged can cause fungal growth inhibition, morphological changes, and fungal cell lysis ( Buitimea-Cantúa *et al.* 2013). ERG11 catalyzes C14-demethylation of lanosterol to 4,4'-dimethyl cholesta-8,14,24-triene-3-β-ol, sterol 14-reductase then reductases to 4,4-dimethylzymosterol, which in turn is converted to zymosterol, and with the help of sterol 8-isomerase is converted into ergosterol. Azole inhibits the synthesis of lanosterol 14α-demethylase (CYP51) ergosterol biosynthesis ( Figure 4) becomes inhibited and damages cell membranes, leading to fungal lysis ( Sanglard *et al.* 2003; Pemán *et al.* 2009; Emami *et al.* 2017; Rosam *et al.* 2021).

Antifungals that damage cell membrane permeability work by binding to ergosterol in the polyene group, inhibiting the synthesis of ergosterol in squalane monooxygenase or epoxidase in the allylamines group, and inhibiting the synthesis of ergosterol in 14-α-demethylase or fungal cytochrome P450 in the azoles group; antifungal destroying cell walls works by inhibiting the synthesis of 1,3-β-glucan by binding to the glucan synthase enzyme which functions to form glucan in the echinocandin group; and antifungal inhibitors of DNA synthesis from fungal cells by inhibiting the synthesis of thymidilate or pyrimidine analogs in flucytosine (5-Fluorocytosine) and mitotic inhibitors in griseofulvin ( Cannon *et al.* 2007; Lewis 2011).

**Phenol**

Phenol (carbolic acid) are secondary metabolites that can be found widely in *Piper* species, but rarely found in algae, fungi, and bacteria; organic compounds with low molecular weight, have one or more substituents hydroxyl group in aromatic phenyl ring, especially benzene, formed from phenylpropanoid or shikimate that produce phenylpropanoids and acetate or malonate polyketide pathway that produce simple phenols or with phenylpropanoids, identified by UV-Vis Spectra and retention times compared with the literature and reference compounds that available ( Waniska 2000; Lattanzio 2013; Ferreira *et al.* 2016).

Phenolic compounds have hydroxyl and carbonyl groups that can interact with fungal cells through hydrogen bonds, thereby increasing protein and cell membranes of pathogen fungal coagulation which will cause the damage and lysis of the fungal cells, and make the next fungal ergosterol growth being anomaly and malformation ( Wagner and Donaldson 2014; Mohamed *et al.* 2017; Silva *et al.* 2018; Januarti *et al.* 2019). Phenolic also had antifungal activities by targeting to destroy the fungal pathogenic effects by inhibiting the fungal dimorphic transition, because dimorphic nature is very important for fungal survival in the host, with making a different morphologies in different conditions or temperatures, both as hyphae (pathogenic) or yeast cells (non-pathogenics) ( Ansari *et al.* 2013).

**Polyphenol**

Polyphenols, known as an antifungal that has been isolated in *Piper* species, are water-soluble, have at least two phenolic rings, usually have 12-16 groups of phenolic hydroxyl at aromatic rings and have a molecular weight from 500 to 3,000 (Da) ( Boulenouar *et al.* 2011; Cheynier 2012). Polyphenols inactivate protein and inhibit enzymes on the surface of bacterial cells; flavonoids form complexes that interfere with the function of the bacterial cell wall, inactivating microbial adhesion, enzymes, and cell protein transport by binding to bacterial extracellular proteins through hydrogen and covalent bonds ( Januarti *et al.* 2019).

**Tannin**

Tannins ([2,3-dihydroxy-5-[[(2 *R*,3 *R*,4 *S*,5 *R*,6 *S*)-3,4,5,6-tetrakis[[3,4-dihydroxy-5-(3,4,5-trihydroxybenzoyl)oxybenzoyl]oxy]oxan-2-yl]methoxycarbonyl]phenyl] 3,4,5-trihydroxybenzoate) are important compounds that have been isolated in the *Piper* species and have beem classified as natural polyphenol groups, water-soluble, a molecular weight of 500-3000 daltons, condensed and hydrolyzed to polymerization that reaches high degrees and have two or three phenolic hydroxyl and carboxyl functional groups on a phenyl ring, known as antimicrobial, against various types of microorganisms, including bacteria, yeasts, fungi, and virus ( Chung *et al.* 1998; de Jesus *et al.* 2012; Kardel *et al.* 2013; Delimont *et al.* 2017; Zhu *et al.* 2019; Ge *et al.* 2020).

Tannins inhibit the chitin growth in the fungi cell wall, which will cause fungal growth inhibition and cell metabolism disruption ( Ridzuan *et al.* 2021) *.* With a high affinity to polysaccharides and proteins, tannins function to form complex compounds with enzymes and substrates, thereby disrupting cell membranes ( Hoste *et al.* 2006; Januarti *et al.* 2019). Plasma membrane and cell wall disruption that being tannin targeted will cause intracellular contents leakage ( Zhu *et al.* 2019). Tannins have the best antifungal activity in *C. albicans* at a concentration above 7.80 mg/L, similar to nystatin, slightly lower than fluconazole, by shoots increasing, substrate and metal ion reduction, germ tube formation inhibition, and wall ultrastructure changing ( Ishida *et al.* 2006).

**Saponins**

Saponins (beta-Escin), sapo in Latin, are one of the important secondary metabolites in the plant, insects, and marine organisms. They are amphiphilic, have surfactant, soap-like foams, and are heat-stable ( Melzig *et al.* 2001; Haralampidis *et al.* 2002; Shi *et al.* 2004), used as herbs and known as antifungal and antibacterial ( Francis *et al.* 2002; Zhang *et al.* 2006). Divided into triterpenoid and steroidal saponins based on their aglycones structure and biochemistry (in plants, the core structures-27 carbon atoms-as furostan (16β, 22-epoxy-cholestan) and spirostan (16α, 22:22α, 26-diepoxy-cholestan), they usually have a hydroxyl group at C-3 position for monodesmosidik, and at C-26 at saponin furastanol bidesmosidik or at C-28 at triterpen bidemosidik, sometimes also reported at C-2, C-15, C-16, C-21, lyobipolar so can affect to lower aqueous surface tension and cell membranes.

Saponins destroy the cell membrane by binding in the cell wall with sterol components so that the pores are formed ( Ridzuan *et al.* 2021). Saponins have hydrophilic molecules and lipid thinning molecules (lipophilic) so that they can make lower cell surface pressure ( Januarti *et al.* 2019). It is reported that 64 μg/ml saponin extract can inhibit *C. albicans* growth and development, by mycelium inhibition, inhibit yeast transition to filamentous, inhibit surface polystyrene adhesion and phospholypase production secretion, and endogenous reactive species oxygen (ROS) induce and the high activity showed by *A. minutiflorum* saponin (minutoside B) against fungal attack ( Barile *et al.* 2007; Jiang *et al.* 2015; Yang *et al.* 2018).

**Flavonoid**

Flavonoids (bioflavonoids), flavus in Latin, yellow powder, low molecular weight, are secondary metabolites that are very useful as antimicrobials by making bacterial damage, contains diphenylpropane (C _6_-C _3_-C _6_), and have a three-carbon bridge with phenyl groups, as the core structure of 2-phenylbenzopyra, less toxic and low cost ( Baum *et al.* 2001; Galeotti *et al.* 2008; Can *et al.* 2015; Kurnia *et al.* 2018; Khalid *et al.* 2019; Herdiyati *et al.* 2020). As an important polyphenol class, flavonoid divided by C-ring oxidation degree, with the three-carbon segment oxidation degree and unsaturation degree, and the major classes are flavonols (3-hydroxy with the different site at OH group of phenolic), flavanones (C-4-keto-group with double-blind of C-2 and C-3), flavanols or flavan-3-ols (C _3_-hydroxyl group and carbon ring that fully saturated), isoflavones (act like phytoestrogens), aurones, chalcones (two aromatic rings by three-unit carbons to make the group of α,β unsaturated carbonyl), and anthocyanidins ( Pourcel *et al.* 2007; Corradini *et al.* 2011; Seleem *et al.* 2017). Flavonoid have activities such as antibacterial, antioxidant, antiinflammation, and antifungal properties by its ability to form complexes with extracellular protein and interfere with the microbial membrane activity because of its lipophilic properties ( Candiracci *et al.* 2011).

Research showed that flavonoid isolated, like 3,4-dihydroxy-5,6,7-trimethoxyflavone, cirsiliol, cirsimaritin, and hispidulin showed antifungal activity to *C. sphaerospermum* ( Alcerito *et al.* 2002). Research also showed that flavonoids in honey have an antifungal activity to *C. albicans* growth inhibition, but not kill the yeasts ( Candiracci *et al.* 2011), prenylated flavanon indicated that high antifungal activity to *Trichophyton* spp with 1.95 g/ml MIC value ( Jin 2019), and flavonoids inhibit the growth of fungal that increased every concentrations level against *Aspergillus niger* van Tieghem, *Aspergillus fumigatus* Fresenius, *Altenaria alternata* (Fr.) Keissler, *Penicillium citrii*, and *Macrophomia phaseolina* (Tassi) Goid ( Kanwal *et al.* 2010).

This literature review summarizes the phytochemicals and antifungal mechanisms of *P. crocatum* that are commonly found worldwide, and also other important activities of this *Piper.* Fungal infections are a problem that often occurs and becomes an ongoing and serious threat to public health ( Kathiravan *et al.* 2012; Ramírez *et al.* 2013). The Polyene amphotericin B is one of the antibiotics that is still often used for the treatment of life-threatening fungal diseases, even though it is known to have toxic side effects ( Odds 2003). Fluconazole are also known to have resistance to *Candida* species due to pressure continuous exposure, drug interactions, and side effects like visual impairment ( Canuto and Rodero 2002; Chen and Sorrell 2007). The increasing use of antifungal treatment causes resistance to antibiotics that are used commonly, even in patients who have never used the drug ( Arif *et al.* 2009; Arendrup 2014). The development of strains that are starting to become resistant to antibiotics from fungal species nowadays is a critical problem that must be addressed immediately in therapeutic problems in society by providing new antifungal agents ( Johnson *et al.* 2004; Jianhua and Hai 2009; Moghadamtousi *et al.* 2014). Natural resources are very important to developing new active molecules and properties, with utilization of natural ingredients as antifungal treatment has a greater level of safety if used appropriately and correctly in terms of dose, time, and method of use ( Vengurlekar *et al.* 2012). Plants have a lot of bioactive secondary metabolites such as flavonoids, terpenoids, alkaloids, tannins, saponins, and other compounds as antifungal agents ( Arif *et al.* 2009). Based on the results of the review, it was found that *P. crocatum* contain compounds that have activity as an antifungal agent so it is expected to become a new antifungal agent that can slowly replace the extensive use of antibiotics. Therefore, new antifungal agents that safer, few side effects, cheaper, easier to get, and more potent against fungal infections are needed.

## Conclusions

Natural products are an important resource in the discovery and development of new medicinal raw materials. *P. crocatum* has antifungal activities that against fungal by its compounds and inhibit ergosterol as a key enzyme in fungal-specific sterols. Damaged cell membrane will cause fungal growth inhibition, morphological changes, and fungal cell lysis. Based on the review data, it is hoped that it can be used as a reference regarding information of new potential bioactive compounds as an alternative treatment for fungal infections by their lanosterol 14 α demethylase CYP51 inhibition effect other than the use of antibiotics or currently used drugs.

## Data availability

No data are associated with this article.
