# Phytochemical profiling of *Piper crocatum* and its antifungal mechanism action as Lanosterol 14 alpha demethylase CYP51 inhibitor: a review

**DOI:** 10.12688/f1000research.125645.3

**Published:** 2023-05-02

**Authors:** Tessa Siswina, Mia Miranti Rustama, Dadan Sumiarsa, Dikdik Kurnia

**Affiliations:** 1 Midwifery, Poltekkes Kemenkes Pontianak, Pontianak, Kalimantan Barat, 78124, Indonesia; 2 Chemistry, Padjadjaran University, Sumedang, Jawa Barat, 45363, Indonesia; 3 Biology, Padjadjaran University, Sumedang, Jawa Barat, 45363, Indonesia

**Keywords:** Piper crocatum, antifungal, phytochemical profiling, lanosterol 14 alpha demethylase, CYP51

## Abstract

Mycoses or fungal infections are a general health problem that often occurs in healthy and immunocompromised people in the community. The development of resistant strains in *Fungi* and the incidence of azole antibiotic resistance in the Asia Pacific which reached 83% become a critical problem nowadays. To control fungal infections, substances and extracts isolated from natural resources, especially in the form of plants as the main sources of drug molecules today, are needed. Especially from *Piperaceae*, which have long been used in India, China, and Korea to treat human ailments in traditional medicine. The purpose of this review is to describe the antifungal mechanism action *from Piper crocatum* and its phytochemical profiling against lanosterol 14a demethylase CYP51. The methods used to search databases from Google Scholar to find the appropriate databases using Preferred Reporting Items for Systematic Reviews and Meta-analyses (PRISMA) Flow Diagram as a clinical information retrieval method. From 1.150.000 results searched by database, there is 73 final results article to review. The review shows that *P. crocatum* contains flavonoids, tannins, terpenes, saponins, polyphenols, eugenol, alkaloids, quinones, chavibetol acetate, glycosides, triterpenoids or steroids, hydroxychavikol, phenolics, glucosides, isoprenoids, and non-protein amino acids. Its antifungal mechanisms in fungal cells occur due to ergosterol, especially lanosterol 14a demethylase (CYP51) inhibition, which is one of the main target sites for antifungal activity because it functions to maintain the integrity and function of cell membranes in *Candida*. *P. crocatum *has an antifungal activity through its phytochemical profiling against fungal by inhibiting the lanosterol 14a demethylase, make damaging cell membranes, fungal growth inhibition, and fungal cell lysis.

## Introduction

Mycoses or fungal infections are a general health problem that often occurs in healthy and immunocompromised people in the community ( Ramírez *et al.* 2013). Fungi are divided into four classes: yeasts, filamentous, dimorphic, and dermatophytes; generally, and ubiquitous in the environment, and become pathogenic when immune cells decrease ( Howard *et al.* 2020). Fungal cells have essentially dynamic structure walls for morphogenesis, pathogenesis, and cell viability and act as a dynamic organelle, and need one-fifth of the yeast genome for cell wall biosynthesis ( Gow *et al.* 2017). The development of resistant strains in fungi and the incidence of azole antibiotic resistance in the Asia Pacific which reached 83% become a critical problem nowadays ( Whaley and Rogers 2016; Whaley *et al.* 2017).

The Azoles are commonly used because cheaper and have a broad spectrum of antimicrobials ( Rosam *et al.* 2021). To control fungal infections, substances, and extracts isolated from natural resources, especially in the form of plants as the main sources of drug molecules today, are needed ( Balouiri *et al.* 2016). Natural products have limited or no side effects on human and animal antifungal activity ( Tabassum and Vidyasagar 2013). Antifungal mechanisms in fungal cells occur due to ergosterol inhibition as a result of 5,6 desaturase (ERG3) downregulation which is the second final step of the ergosterol biosynthetic pathway ( Alizadeh *et al.* 2017). Ergosterol at the fungal plasma membrane is the most common sterol and binding at lanosterol 14α demethylase, an ergosterol-specific enzyme that can cause lanosterol demethylation ( Loeffler and Stevens 2003; Ashley *et al.* 2006; Emami *et al.* 2017).

*Piperaceae* plant extracts have long been used in India, China, and Korea to treat human ailments in traditional medicine ( Jeon *et al.* 2019). The part of the *Piperaceae* family which has large species of up to 1000 is the *Piper* genus ( Durant-Archibold *et al.* 2018). *Piper* can be found in temperate regions with tropical and sub-tropical ( Lima *et al.* 2020). Indonesia is located on the equator, which has a tropical climate with high humidity and many natural and biological resources ( Puspita *et al.* 2018). The seeds and leaves of *Piper* species are often cultivated and consumed for various diseases treatment such as antifungal, antibacterial, and disinfectant effects ( Astuti *et al.* 2014; Mgbeahuruike *et al.* 2017).

Isolation of several secondary metabolites of *Piper* species shows that therapeutically molecules like lignans, flavones, alkaloids, unsaturated amides, long and short-chain esters, aristolactams, monoterpenes, sesquiterpenes, ketones, aldehydes, arylpropanoids, chalcones, propenylphenols, and amide alkaloids as a typical constituent ( Gutierrez *et al.* 2013). However, no tests were found on specific compounds for antibacterial activity from *Piper* ( Barh *et al.* 2013). Based on the literature, antifungal compounds are classified into flavonoids, amides, acid derivatives, lignans, prenylated benzoic, cyclopentanedione, butenolides, and phenylpropanoids ( Xu and Li 2011). Of the various *Piper* species, the main constituent is an amide, which is classified as aristolactams, open-chain alkamides, amides with pyrrolidine, 4,5-dioxoaporphines, Piperidine, and Piperidone groups, ceramides, cyclohexanamid, and cyclobutanamide ( Do Nascimento *et al.* 2012). In this review, we summarize the antifungal activity properties, structural studies, and bio-mechanism of *P. crocatum*, commonly known as Red Betel leaf, which is found worldwide, against lanosterol 14 α demethylase CYP51 in fungi. This review is expected to allow us to find new alternative antifungal treatment agents from natural resources based on their active chemical compounds and activities to cure fungal infections, thus reducing the extensive and inappropriate use of antibiotics for antifungal treatment.

## Methods

**Figure 1.**
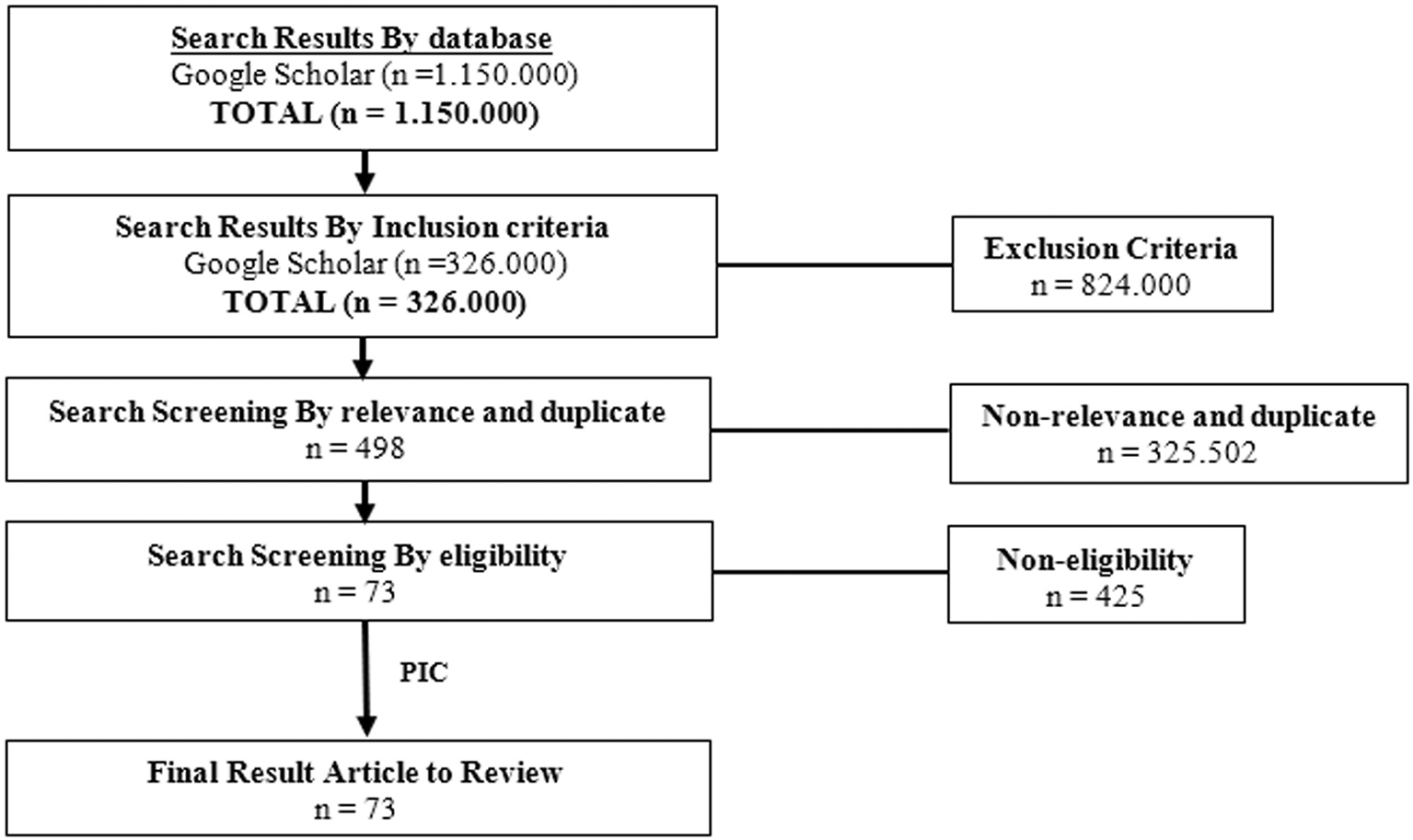
The flowchart of literature reviews of *P. crocatum.*

The author searches databases from Google Scholar to find the appropriate databases using the Preferred Reporting Items for Systematic Reviews and Meta-analyses (PRISMA) Flow Diagram as a clinical information retrieval method. The search screening occurred through four stages. The first stage was screening by the keywords, the second stage was screening by the criteria, the third stage was screening by relevance and duplicates, and the fourth stage was screening by eligibility. The keyword of this search was ‘ *Piper’* which yielded 1,150,000 results. The second step was screening by inclusion criteria of the keyword ‘ *Piper’* that reported articles published from 2003 until 2022, which yielded 326,000 results. For the third step, screening by relevance and duplicate with the keyword of this search is ‘ *Piper crocatum’* AND ‘antifungal’, which yielded about 498 results. The final screening results included 73 articles to review. This search was conducted from February to May 2022. The criteria for this research were clinical trials in animal testing and humans, books, laboratory tests, case studies, article reviews, systematic reviews, narrative reviews, and meta-analyses. The study was conducted with a true-experimental (Double-Blind RCT), quasi-experimental, study protocol, or pilot study. Articles were published in English. The flow chart of the Literature Review showed in Figure 1.

## Results and discussion

### Ethno-botany and ethnopharmacology of *P. crocatum*

*P. crocatum*, used as spices, vegetables, and components in some ethnic’s ceremonial, and medicinal herbs by the local community in Southeast Asian Countries especially Indonesia to treat candidiasis, hypertension, hepatitis, diabetes mellitus, kidney failure, cholesterol, prevent stroke, breast cancer, hemorrhoids, inflammation, coughing up blood, nose bleed, vaginal discharge, and tuberculosis disease ( Astuti *et al.* 2014; Lely *et al.* 2021). *P. crocatum*, a plant native from Peru then spread to several regions in the world, and grows in temperate climates (tropical and subtropical) ( Lima *et al.* 2020). Red betel is a shrub that consists of trunked, 5-10 cm continuous branches, with each segment having roots growing ( Safithri and Fahma 2008). The stems of red betel are round, purplish-green heart-shaped leaves, have no flowers, green with grayish-white tapered ends, special aroma, and are very bitter ( Hermiati *et al.* 2013).

The taxonomy of *P. crocatum* is as follows ( Suri *et al.* 2021):

Kingdom: Plantae

Subkingdom: Tracheobionta

Division: Magnoliophyta

Class: Magnoliopsida

Order: Piperales

Family: Piperaceae

Genus: Pipers

Species: *Piper crocatum* Ruiz&Pav

According to ethnopharmacology data, *P. crocatum* leaves have anxiolytic, analgesic, anti-inflammatory, vasodilatory, immunomodulatory, antimicrobial, antifungal, antitumor, antibacterial, anti-cariogenic, antifungal, anti larva, anti protozoa, anti filaria, antiallergic, antidiabetic, antihelmintic, stress relieves, antihyperglycemic, antibiotics, platelet inhibitors, and antioxidants ( Bezerra *et al.* 2008; Rodrigues *et al.* 2009; Fadlilah 2015; Fatmawaty *et al.* 2019; Madhumita *et al.* 2020).

### The phytochemical of *P. crocatum*

Isolated *P. crocatum* from 112 species contains 677 different compounds, consisting of 190 alkaloids or amides, 97 terpenes, 70 neolignans, 49 lignans, 39 phenylpropanoids, 18 kavapyrones, 17 chalcones or dihydrochalcones, 16 flavones, 15 steroids, 6 flavanones, 4 cinnamylidone butenolides or piperolides, and 146 other compounds ( Saputra *et al.* 2016). Isolation of several secondary metabolites of *P. crocatum* contains flavonoids, tannins, terpenes, saponins, polyphenols, eugenol ( **1**), alkaloids, quinones ( **2**), chavibetol acetate, glycosides ( **3**), triterpenoids ( **4**) or steroids, hydroxychavikol ( **5**), phenolics, glucosides ( **6**), isoprenoids, and non-protein amino acids ( Erviana 2011; Gutierrez *et al.* 2013; Saputra *et al.* 2016; Li *et al.* 2019; Lister *et al.* 2020; Safithri *et al.* 2020).

**Figure 2.**
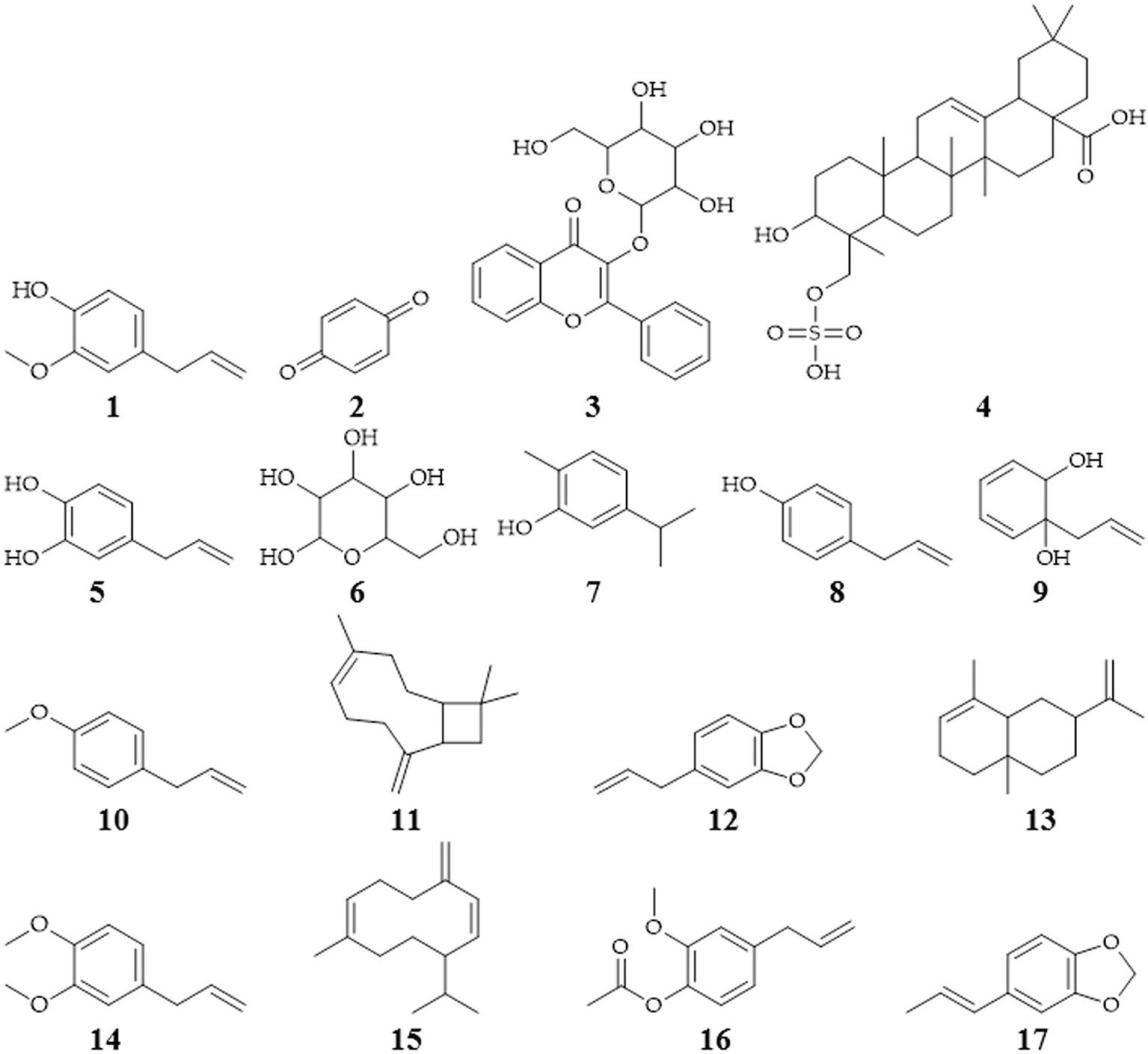
Chemical structures of *P. crocatum* ( Saputra *et al. *2016; Fatmawaty *et al.* 2019; Lister *et al.* 2020).

The bioactive compounds of *P. crocatum* are as follows in Table 1 and Figure 2.

**Table 1.** The bioactive compounds of *P. crocatum*.

Extract type	Chemical compounds				
	Used ether 40-60°C, chloroforms, ethanol, and water solvent ( Suri *et al.* 2021)	Used ethanol solvent ( Fatmawaty, Anggreni, Fadhil, & Prasasty 2019)	Used methanol Solvent ( Januarti, Wijayanti, Wahyuningsih, & Nisa 2019)	Used methanol solvent ( Safithri & Fahma 2008)	Used methanol extract ( Li, Yang, Kim, & Li 2019)
Red Betel leaf extract	Alkaloid	Alkaloid	Saponin	Alkaloid	Pipercroside A
	Carbohydrate	Glycosides	Tannin	Flavonoid	Pipercroside B
	Water	Saponin	Phenol	Tannin	2,5-Dimethoxy-3-glucopyranosylcinnamic alcohol
	Tannin	Tannin	Flavonoid		Cimidahurinin
	Phenol	Triterpenoid/Steroid			Erigeside II
	Flavonoid	Flavonoid			Syringin
	Essential oil	Essential oil			β-Phenylethyl β- *D*-glucoside
					Methyl salicylate 2- *O*-β- *D*-Glucopyranoside
					Icariside D1
					4-Hydroxybenzoic acid β- *D*-glucosyl ester
					Benzyl β- *D*-Glucoside
					Phenylmethyl 6- *O*-α- *L*-Arabinofuranosyl-β- *D*-glucopyranoside
Red Betel leaf essential oil (water distillation, sodium sulfate dryer)	Carvacrol (7)				
	Eugenol ( **1**) 28.44%				
	Chavicol ( **8**)				
	Allylcatechol ( **9**)				
	Cinema				
	Estragole ( **10**)				
	Caryophyllene ( **11**)				
	Pcymenedaneugenol Metil eter-19				
	Safrole ( **12**) 27.48%				
	Selinene ( **13**) 7.32%				
	Methyl eugenol ( **14**) 1.46%				
	Germacrene D ( **15**) 0.91%				
	Eugenyl Acetate ( **16**) 1.72%				
	Isosafrole ( **17**) 1.62%				

The heated leaves are often used to relieve asthma, sore throats, coughs, and vaginal discharge, while the essential oil is used as catarrh and diphtheria inhalation and mouthwash, with antifungal activity ability against *Candida sphaerospermum* and *Candida cladospoiroides* with strong MIC of 10 μg/ml ( Safithri *et al.* 2020; Suri *et al.* 2021). The antimicrobial compounds include phenolics and flavonoids in *P. crocatum* by its hydroxyl group at 5 positions causing inner and outer bacterial cell membrane fluidity reduction ( Puspita *et al.* 2018). Alkaloid in *P. crocatum* by its aromatic substitution, carbon rings, and oxidation nature caused inhibition of bacterial growth and cell lysis. Tannins also have antibacterial activity by slowing the fungal cell's growth, and shrinking cell membranes thereby limiting the development of cell membrane synthesis, distorting permeability, breakdown, and cell lysis while saponins can dilute lipids (lipophilic) and then reducing cell surface pressure with their ability to attract water (hydrophilic) and caused cell damage. Terpenoids in *P. crocatum* cause permeability decreased and rupture of cell membranes so the nutrients and enzymes leave the cytoplasm, decrease metabolism, reduce ATP production, and inhibit bacterial growth and reproduction ( Cowan 1999; Rinanda *et al.* 2012).

### Test methods and antifungal properties of *P. crocatum*

*P. crocatum* has been tested on *C. albicans* with agar diffusion method ( Kusuma *et al.* 2017), MIC determination with microdilution method, and MBC determination by the medium surface of Mueller Hinton Agar, and shows that extract of ethanol indicates antifungal activity, MIC value 1.25-2.5% w/v, and MBC value 0.75 min ( Kusuma *et al.* 2017). The study shows about ten (10) known compounds (2,5-dimethoxy-3-glucopyranosylcinnamic alcohol, cimidahurinin, erigeside II, syringin, β-phenylethyl β-D-glucoside, methyl salicylate 2- *O*-β-D-glucopuranoside, icariside D1, 4-hydroxybenzoic acid β-D-glucosyl ester, benzyl β-D-glucoside, and phenylmethyl 6- *O*-α-L-arabinofuranosyl-β-D-glucopyranoside), and two new phenolic glucosides (Pipercroside A and B) that isolated from MeOH extract of *P. crocatum* elucidated by spectroscopy 1D and 2D NMR, HR-ESI-MS analysis also reports erigeside II have the best antifungal activity with IC _50_ value as 58.5 ( Li *et al.* 2019).

Polyphenol inactivates protein and inhibits enzymes on the surface of bacterial cells; flavonoids form complexes that interfere with the function of the bacterial cell wall, inactivating microbial adhesion, enzymes, and cell protein transport by binding to bacterial extracellular proteins through hydrogen and covalent bonds; saponins have hydrophilic molecules and lipid thinning molecules (lipophilic) so that they can make lower cell surface pressure; tannins functions to form complex compounds with enzymes and substrates, thereby disrupting cell membranes, and phenol has hydroxyl and carbonyl groups that can interact with fungal cells through hydrogen bonds, thereby increasing protein coagulation and fungal cell membranes which will cause fungal cells to lyse ( Januarti *et al.* 2019). Inhibition of fungal activity can be done by bothering cell membranes, the activity of enzymes, and fungi genetic mechanisms ( Ejele *et al.* 2012).

### Antifungal properties and structure

Treatment of vaginal discharge due to *Candida* gave the best response to a combination of the intravaginal vulva and topical therapy ( Mitchell 2004). Antifungals for the treatment of vaginal discharge caused by *C. albicans* are fluorinated pyrimidine cytosine (5-FC) which targets RNA synthesis and DNA replication, polyenes which affect the integrity of cell membranes, azoles which affect the target of the ergosterol biosynthetic pathway, and echinocandins which affect cell wall biosynthesis, while the use of broad-spectrum antibiotics increases cases of immunocompromise ( Yücesoy and Marol 2003; Sendid *et al.* 2007).

Antifungals that damage cell membrane permeability work by binding to ergosterol in the polyene group, inhibiting the synthesis of ergosterol in squalene monooxygenase or epoxidase in the allylamines group, and inhibiting the synthesis of ergosterol in 14-α-demethylase or fungal cytochrome P450 in the azoles group; antifungal destroying cell walls works by inhibiting the synthesis of 1,3-β-glucan by binding to the glucan synthase enzyme which functions to form glucan in the echinocandin group; and antifungal inhibitors of DNA synthesis from fungal cells by inhibiting the synthesis of thymidylate or pyrimidine analogs in flucytosine (5-Fluorocytosine) and mitotic inhibitors in griseofulvin ( Cannon *et al.* 2007; Lewis 2011).

**Figure 3.**
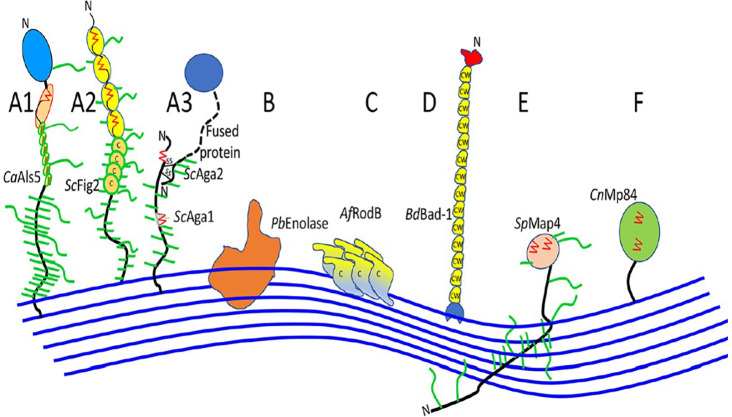
An illustration of cell wall association in fungal adhesins (the blue line indicates the cell wall which is composed of glucan polymers; Ca, *C. albicans*; Sc, *S. cerevisiae*; Pb, *P. braziliensis*; Af, *A. fumigatus*; Bd, *B. dermatitidis*; Sp, *S. pombe*; Cn, *C. neoformans; A, adhesins; B-E, modes of cell wall attachment; A1 and A3, have discrete ligand binding domains; A2 doesn’t have discrete ligand binding domains, Cys-rich sequences in ScFig2; C, Cys-rich sequences in AfRodB; D, Cys/Trp rich domains in Bd/Bad-1; F, attached by modified GPI anchors to the cell wall*) ( Lipke 2018).

Fungal cell walls, fungal-specific, serve as protection from harmful environments and are aggressive because of its toxic and hydrolytic molecules. Ninety percent consist of polysaccharides, with *Saccharomyces* and *Candida* subphylum as the well-characterized fungal adhesins ( Figure 3) ( Latgé 2007). The core of the central fungal cell wall consists of chitin that is linked to branched β-1,3-glucan, combined with galactomannan, galactosamynoglycan, and β-1,3-1,4-glucan in *A. fumigatus* and β-1,6-glucan in *C. albicans* ( Adams 2004; Latgé 2010). Chitin, with a weight of 1-2% from dry cell wall yeast, is an important structure that consists of a homopolymer of β-1,4-linked *N*-acetylglucosamine that is long and linear, while disrupted chitin synthesis will cause the fungal cell wall to be lysis and unstable in osmotic ( Bowman and Free 2006).

**Figure 4.**
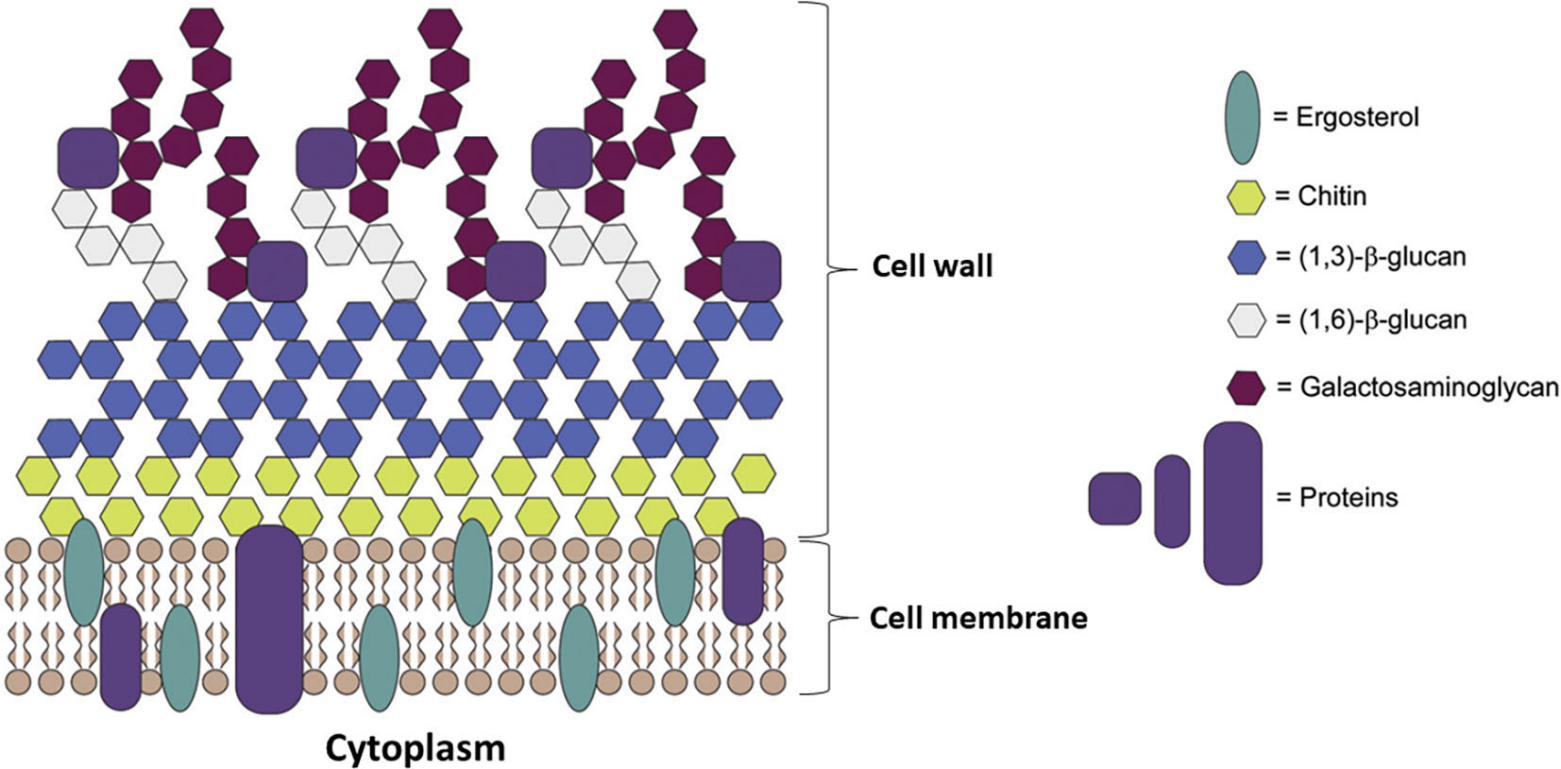
The cell wall and membrane of Candida ( Emami *et al.* 2017; Rosam *et al.* 2021).

Ergosterol is a bitopian endoplasmic reticulum protein, which spans the entire length of the lipid bilayer ( Figure 4) ( Emami *et al.* 2017; Rosam *et al.* 2021). Ergosterol is a key enzyme in fungal-specific sterols, cytochrome P450 enzyme in fungi derived from *S. cerevisiae*, belonging to the CYP51 (lanosterol 14-α-demethylase) family. The biosynthesis inhibition of ergosterol will be caused by intermediates toxic sterol accumulation (14-α-methyl-3,6-diol) by ERG3. By binding the nitrogen atom containing heterocyclic moiety in the core ring to the iron atom of the heme domain group in the active site and preventing the formation of lanosterol demethylation, the cell membranes will get damaged, and lysis ( Arthington-Skaggs *et al.* 1999; Flowers *et al.* 2015). Ergosterol biosynthesis is one of the main target sites for antifungal activity because ergosterol functions to maintain the integrity and function of cell membranes in *Candida* so it can cause fungistatic effects if there is inhibition of lanosterol 14α demethylase ( Shareef *et al.* 2019; Kumar *et al.* 2020).

**Figure 5.**
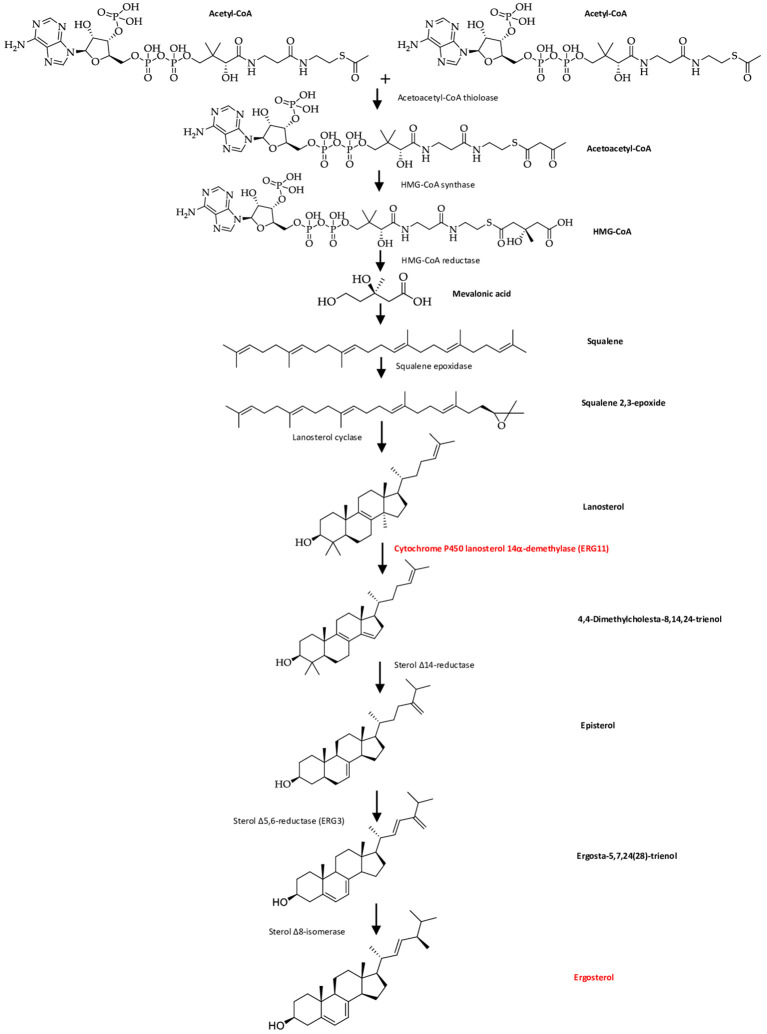
The diagram of ergosterol formation in fungal cells ( Emami *et al.* 2017).

**Figure 6.**
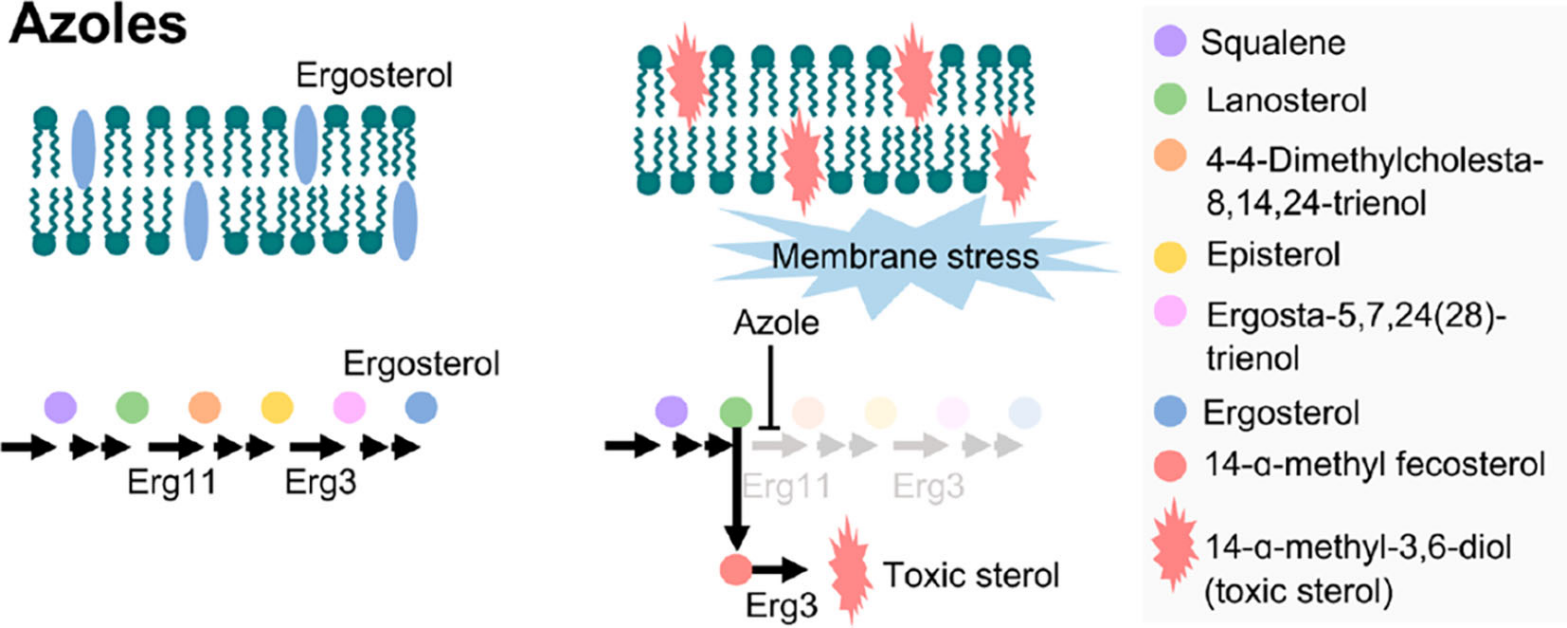
The fungal lysis process ( Lee *et al.* 2021).

The accumulation of toxic sterol and depletion of ergosterol causes inhibition of cell growth and division, then increases pressure on the cell wall so that the cell becomes damaged ( Robbins *et al.* 2016). Fungal cell walls that have been damaged can cause fungal growth inhibition, morphological changes, and fungal cell lysis ( Buitimea-Cantúa *et al.* 2013). ERG11, the major target in the fungal membrane that is absent in the host cell membrane catalyzes C14-demethylation of lanosterol to 4,4′-dimethyl cholesta-8,14,24-triene-3-β-ol, sterol 14-reductase then reductases to episterol, which in turn is converted to ergosta-5,7,24(28)-trienol by sterol 5,6 desaturase (ERG3), and with sterol 8-isomerase converted it into ergosterol ( Figure 5). The fungistatic mechanism by inhibiting lanosterol 14α demethylase (encoded by ERG11), which leads to a block in ergosterol synthesis and the accumulation of toxic sterol intermediates, including 14-α-methyl-3,6-diol produced by Erg3 ( Lee *et al.* 2021). This toxic sterol gave the membrane cell heavy stress and impairs cell membrane permeability, thereby ergosterol biosynthesis becomes inhibited and cell membranes get damaged, leading to fungal lysis ( Figure 6) ( Sanglard *et al.* 2003; Pemán *et al.* 2009; Emami *et al.* 2017; Rosam *et al.* 2021).

**Phenol**

Phenol (carbolic acid) are secondary metabolites that can be found widely in *Piper* species, but rarely found in algae, fungi, and bacteria; organic compounds with low molecular weight, have one or more substituents hydroxyl group in aromatic phenyl ring, especially benzene, formed from phenylpropanoid or shikimate that produce phenylpropanoids and acetate or malonate polyketide pathway that produce simple phenols or with phenylpropanoids, identified by UV-Vis Spectra and retention times compared with the literature and reference compounds that available ( Waniska 2000; Lattanzio 2013; Ferreira *et al.* 2016).

Phenolic compounds have hydroxyl and carbonyl groups that can interact with fungal cells through hydrogen bonds, thereby increasing protein and cell membranes of pathogen fungal coagulation which will cause the damage and lysis of the fungal cells, and make the next fungal ergosterol growth anomaly and malformation ( Wagner and Donaldson 2014; Mohamed *et al.* 2017; Silva *et al.* 2018). Phenolic also had antifungal activities by targeting to destroy the fungal pathogenic effects by inhibiting the fungal dimorphic transition, because dimorphic nature is very important for fungal survival in the host, with making different morphology in different conditions or temperatures, both as hyphae (pathogenic) or yeast cells (non-pathogenic) ( Ansari *et al.* 2013).

**Polyphenol**

Polyphenols, known as an antifungal that has been isolated in *Piper* species, are water-soluble, have at least two phenolic rings, usually have 12-16 groups of phenolic hydroxyl at aromatic rings, and have a molecular weight from 500 to 3,000 (Da) ( Boulenouar *et al.* 2011; Cheynier 2012). Polyphenols inactivate protein and inhibit enzymes on the surface of bacterial cells; flavonoids form complexes that interfere with the function of the bacterial cell wall, inactivating microbial adhesion, enzymes, and cell protein transport by binding to bacterial extracellular proteins through hydrogen and covalent bonds ( Januarti *et al.* 2019).

**Tannin**

Tannins ([2,3-dihydroxy-5-[[(2 *R*,3 *R*,4 *S*,5 *R*,6 *S*)-3,4,5,6-tetrakis[[3,4-dihydroxy-5-(3,4,5-trihydroxybenzoyl)oxybenzoyl]oxy]oxan-2-yl]methoxycarbonyl]phenyl] 3,4,5-trihydroxybenzoate) are important compounds that have been isolated in the *Piper* species and have been classified as natural polyphenol groups, water-soluble, a molecular weight of 500-3000 daltons, condensed and hydrolyzed to polymerization that reaches high degrees and have two or three phenolic hydroxyl and carboxyl functional groups on a phenyl ring, known as antimicrobial, against various types of microorganisms, including bacteria, yeasts, fungi, and virus ( Chung *et al.* 1998; de Jesus *et al.* 2012; Kardel *et al.* 2013; Delimont *et al.* 2017; Ge *et al.* 2020).

Tannins inhibit the chitin growth in the fungi cell wall, which will cause fungal growth inhibition and cell metabolism disruption ( Ridzuan *et al.* 2021) *.* With a high affinity to polysaccharides and proteins, tannins function to form complex compounds with enzymes and substrates, thereby disrupting cell membranes ( Hoste *et al.* 2006). Plasma membrane and cell wall disruption that being tannin targeted will cause intracellular contents leakage ( Zhu *et al.* 2019). Tannins have the best antifungal activity in *C. albicans* at a concentration above 7.80 mg/L, similar to nystatin, slightly lower than fluconazole, by shoots increasing, substrate and metal ion reduction, germ tube formation inhibition, and wall ultrastructure changing ( Ishida *et al.* 2006).

**Saponins**

Saponins (beta-Escin), sapo in Latin, are one of the important secondary metabolites in plant, insects, and marine organisms. They are amphiphilic, have surfactant, soap-like foams, and are heat-stable ( Melzig *et al.* 2001; Haralampidis *et al.* 2002; Shi *et al.* 2004), used as herbs and known as antifungal and antibacterial ( Francis *et al.* 2002; Zhang *et al.* 2006). Divided into triterpenoid and steroidal saponins based on their aglycones structure and biochemistry (in plants, the core structures-27 carbon atoms-as furostan (16β, 22-epoxy-cholestan) and spirostan (16α, 22:22α, 26-diepoxy-cholestan), they usually have a hydroxyl group at C-3 position for monodesmosidik, and at C-26 at saponin furastanol bidesmosidik or C-28 at triterpene bidemosidik, sometimes also reported at C-2, C-15, C-16, C-21, lyobipolar so can affect to lower aqueous surface tension and cell membranes.

Saponins destroy the cell membrane by binding in the cell wall with sterol components so that the pores are formed ( Ridzuan *et al.* 2021). Saponins have hydrophilic molecules and lipid thinning molecules (lipophilic) so that they can make lower cell surface pressure ( Januarti *et al.* 2019). It is reported that 64 μg/ml saponin extract can inhibit *C. albicans* growth and development, by mycelium inhibition, inhibits yeast transition to filamentous, inhibits surface polystyrene adhesion and phospholipase production secretion, and endogenous reactive species oxygen (ROS) induce and the high activity showed by *A. minutiflorum* saponin (minutoside B) against fungal attack ( Barile *et al.* 2007; Jiang *et al.* 2015; Yang *et al.* 2018).

**Flavonoid**

Flavonoids (bioflavonoids), flavus in Latin, yellow powder, low molecular weight, are secondary metabolites that are very useful as antimicrobials by making bacterial damage, contain diphenylpropane (C _6_-C _3_-C _6_), and have a three-carbon bridge with phenyl groups, as the core structure of 2-phenylbenzopyra, less toxic and low cost ( Baum *et al.* 2001; Galeotti *et al.* 2008; Can *et al.* 2015; Kurnia *et al.* 2018; Khalid *et al.* 2019; Herdiyati *et al.* 2020). As an important polyphenol class, flavonoids divided by C-ring oxidation degree, with the three-carbon segment oxidation degree and unsaturation degree, and the major classes are flavonols (3-hydroxy with the different site at OH group of phenolic), flavanones (C-4-keto-group with double-blind of C-2 and C-3), flavanols or flavan-3-ols (C _3_-hydroxyl group and carbon ring that fully saturated), isoflavones (act like phytoestrogens), aurones, chalcones (two aromatic rings by three-unit carbons to make the group of α,β unsaturated carbonyl), and anthocyanidins ( Pourcel *et al.* 2007; Corradini *et al.* 2011; Seleem *et al.* 2017). Flavonoids have activities such as antibacterial, antioxidant, anti-inflammation, and antifungal properties through its ability to form complexes with extracellular proteins and interfere with microbial membrane activity because of its lipophilic properties ( Candiracci *et al.* 2011).

Research showed that flavonoids isolated, like 3,4-dihydroxy-5,6,7-trimethoxyflavone, cirsiliol, cirsimaritin, and hispidulin showed antifungal activity to *C. sphaerospermum* ( Alcerito *et al.* 2002). Research also showed that flavonoids in honey have an antifungal activity to *C. albicans* growth inhibition, but not kill the yeasts ( Candiracci *et al.* 2011), prenylated flavanones indicated that high antifungal activity to *Trichophyton* spp with 1.95 g/ml MIC value ( Jin 2019), and flavonoids inhibit the growth of fungal that increased every concentrations level against *Aspergillus niger* van Tieghem, *Aspergillus fumigatus* Fresenius, *Altenaria alternata* (Fr.) Keissler, *Penicillium citrii*, and *Macrophomia phaseolina* (Tassi) Goid ( Kanwal *et al.* 2010).

This literature review summarizes the phytochemicals and antifungal mechanisms of *P. crocatum* that are commonly found worldwide, as also other important activities of this *Piper.* Fungal infections are a problem that often occurs and becomes an ongoing and serious threat to public health ( Kathiravan *et al.* 2012). Polyene amphotericin B is one of the antibiotics that still often used for the treatment of life-threatening fungal diseases, even though it is known to have toxic side effects ( Odds 2003). Fluconazole is also known to have resistance to *Candida* species due to pressure continuous exposure, drug interactions, and side effects like visual impairment ( Canuto and Rodero 2002; Chen and Sorrell 2007). The increasing use of antifungal treatment causes resistance to antibiotics that are used commonly, even in patients who have never used the drug ( Arif *et al.* 2009; Arendrup 2014). The development of strains that are starting to become resistant to antibiotics from fungal species nowadays is a critical problem that must be addressed immediately in therapeutic problems in society by providing new antifungal agents ( Johnson *et al.* 2004; Jianhua and Hai 2009; Moghadamtousi *et al.* 2014). Natural resources are very important to developing new active molecules and properties, with the utilization of natural ingredients as antifungal treatment having a greater level of safety if used appropriately and correctly in terms of dose, time, and method of use ( Vengurlekar *et al.* 2012). Plants have a lot of bioactive secondary metabolites such as flavonoids, terpenoids, alkaloids, tannins, saponins, and other compounds as antifungal agents ( Arif *et al.* 2009). Due to the high rate of antibiotic resistance in the treatment of antifungal because of long-term use, new antifungal treatment agents are needed. The new antifungal should be safer, have minimal side effects, be cheaper, easier to get, and more potent against fungal infections. Based on the results of the review, it was found that *P. crocatum* contains compounds that have antifungal activity. By its secondary metabolites, *P. crocatum* has the opportunity to become a new antifungal agent as an alternative non-pharmacological antifungal treatment.

## Conclusions

Natural products are important resources in the discovery and development of new medicinal raw materials. *P. crocatum* has antifungal activities that are against fungal by its compounds and inhibit ergosterol as a key enzyme in fungal-specific sterols. The inhibition of ergosterol can be induced by the inhibition of lanosterol 14 α demethylase in the biosynthesis which will cause integrity and function damage to fungal cell membranes. Damaged cell membranes will cause fungal growth inhibition, morphological changes, and fungal cell lysis. Based on the review data, it is hoped that it can be used as a reference regarding information of new potential bioactive compounds as an alternative treatment for fungal infections by their lanosterol 14 α demethylase CYP51 inhibition effect other than the use of antibiotics or currently used drugs.

## Data availability

No data are associated with this article.
