# Supplementary material for: Phytochemical profiling of Piper crocatum and its antifungal mechanism action as Lanosterol 14 alpha demethylase CYP51 inhibitor: a review
Source: F1000Res. 2023 May 2;11:1115. Originally published 2022 Sep 28. [Version 3] doi: 10.12688/f1000research.125645.3 (PMC10157293; doi:10.12688/f1000research.125645.3)
Supplement: Peer review history

# Peer review: Reviewer response for version 3

**DOI:** 10.5256/f1000research.147354.r171647

**Published:** 2023-05-03

**Authors:** Anna Safitri

**Affiliations:** 1 Research Center of Smart Molecule of Natural Genetics Resource, Universitas Brawijaya, Malang, East Java, Indonesia; 2 Department of Chemistry, Faculty of Mathematics and Natural Sciences, Universitas Brawijaya, Malang, East Java, Indonesia

I have received the following manuscript for review from F1000Research: "Phytochemical profiling of Piper crocatum and its antifungal mechanism action as Lanosterol 14 alpha demethylase CYP51 inhibitor". This is a revised version from my suggestions.

 I confirm that the authors have been revised the manuscript based on my comments and suggestions.

Is the review written in accessible language?

Partly

Are all factual statements correct and adequately supported by citations?

Yes

Are the conclusions drawn appropriate in the context of the current research literature?

Partly

Is the topic of the review discussed comprehensively in the context of the current literature?

Yes

Reviewer Expertise:

Chemistry, Biochemistry

I confirm that I have read this submission and believe that I have an appropriate level of expertise to confirm that it is of an acceptable scientific standard.

# Peer review: Reviewer response for version 2

**DOI:** 10.5256/f1000research.145688.r169051

**Published:** 2023-04-21

**Authors:** Anna Safitri

**Affiliations:** 1 Research Center of Smart Molecule of Natural Genetics Resource, Universitas Brawijaya, Malang, East Java, Indonesia; 2 Department of Chemistry, Faculty of Mathematics and Natural Sciences, Universitas Brawijaya, Malang, East Java, Indonesia

I have received the following manuscript for review from F1000Research: "Phytochemical profiling of *Piper crocatum* and its antifungal mechanism action as Lanosterol 14 alpha demethylase CYP51 inhibitor".

**General comments:**

- I think overall the manuscript need a proof reading for the English language and grammar.

The following sections required revisions:

- Results and Discussion; and Conclusion

Results and Discussion

- Table 1:
  - The name of the species *P. crocatum* suddenly was changed into betel plant? In the introduction section can be mentioned that betel plant is a common name for *P. crocatum.*
- The betel leaf extract was extracted using which solvent (alcohol or other organic solvents?)
- I suggest the bioactive compounds contained in *P. crocatum* should be derived from more than 1 references.
- A sentence below Figure 2 here: *"The bioactive compound of P. crocatum as follows Table 1 and Figure 2"*.
  This sentence is missing a verb (grammatically incorrect).
- Figure 3 caption: *"Illustrating of cell wall…"*, should be ' **an** Illustration of cell wall…'.
  Also there are capital letters in Figure 3: A1, A2, A3, B, C, D, E, F, and these are not mentioned in the Figure caption. What are those?
- A sentence here above Figure 3:
  - *"Ergosterol is a key enzyme in fungal-specific sterols, cytochrome P450 enzyme in fungi derived from S. cerevisiae, belonging to the CYP51 (lanosterol 14-α-demethylase) family, so with inhibition of biosynthesis in ergosterol, caused intermediates toxic sterol accumulation (14-α-methyl-3,6-diol) by ERG3, by binding the nitrogen atom containing heterocyclic moiety in the core ring to the iron atom of the heme domain group in the active site and preventing the formation of lanosterol demethylation, will damage cell membranes (Arthington-Skaggs et al.1999; Flowers et al. 2015)."*
  This sentence is too long and unclear. Authors should break this sentence into 2-3 sentences.
- All the phytochemical content in *P. crocatum*, do they have activities as Lanosterol 14 alpha demethylase CYP51 inhibitor? This is because in the explanations of phytochemicals like polyphenols, tannins, saponins, it seems they don’t have activities as inhibitor for demethylase CYP51. Or these are different activities as anti fungi?
- These sentences before conclusion section:
  - *"Based on the results of the review, it was found that P. crocatum contain compounds that have activity as an antifungal agent so it is expected to become a new antifungal agent that can slowly replace the extensive use of antibiotics. Therefore, new antifungal agents that safer, few side effects, cheaper, easier to get, and more potent against fungal infections are needed."*
  I think these sentence need to be revised, since the sentences are unclear in particular the last sentence.

Conclusions

- The conclusion section need to be revised since there are grammatical error and unclear. For example: I *Natural products are an important resource…"*, should be “Natural products are important resources…"
- Conclusion should emphasize the antifungal activities of *P. crocatum*. Does *P. crocatum *have antifungal activities by inhibiting ergosterol synthesis or as a lanosterol 14 α demethylase CYP51 inhibitor?

Is the review written in accessible language?

Partly

Are all factual statements correct and adequately supported by citations?

Yes

Are the conclusions drawn appropriate in the context of the current research literature?

Partly

Is the topic of the review discussed comprehensively in the context of the current literature?

Yes

Reviewer Expertise:

Chemistry, Biochemistry

I confirm that I have read this submission and believe that I have an appropriate level of expertise to confirm that it is of an acceptable scientific standard, however I have significant reservations, as outlined above.

# Response

**Published:** 2023-04-22

**Authors:** Dikdik Kurnia

**Affiliations:** Universitas Padjadjaran, Indonesia

**Evaluation for authors**

 1. General comments

**Answer**:

**Thank you for your suggestion**.

 The authors have revised and followed the suggestions.

 2. Table 1

 The name of the species P. crocatum suddenly was changed into betel plant? In the introduction section can be mentioned that betel plant is a common name for P. crocatum

**Answer**:

**Thank you for your suggestion**.

 The authors have added the common name of P. crocatum as Red Betel plant in the introduction. 

 3. Table 1

 The Red Betel leaf extract was extracted using which solvent (alcohol or other organic solvents?)

**Answer**:

**Thank you for the correction and suggestion.**

 The authors have revised and added an explanation about this sentence in the narrative.

 4. Table 1

 I suggest the bioactive compounds contained in P. crocatum should be derived from more than 1 references

**Answer**:

**Thank you for your correction and suggestion**.

 The authors have added 4 (four) new references to P. crocatum bioactive compounds.

 5. Table 1

 A sentence below Figure 2 here: "The bioactive compound of P. crocatum as follows Table 1 and Figure 2". This sentence is missing a verb (grammatically incorrect).

**Answer**:

**Thank you for your correction and suggestion**.

 The authors have revised the grammar in the sentence.

 6. Figure 3

 "Illustrating of cell wall…", should be ' **an** Illustration of cell wall…'.

 Also there are capital letters in Figure 3: A1, A2, A3, B, C, D, E, F, and these are not mentioned in the Figure caption. What are those?

**Answer**:

**Thank you for your correction and suggestion**.

 The authors have revised the sentence and added the explanation in the figure title.

 7. Narrative in Figure 3

 This sentence is too long and unclear. Authors should break this sentence into 2-3 sentences.

**Answer**:

**Thank you for your correction and suggestion**.

 The authors have revised this sentence into 3 sentences as the reviewer suggested.

 8. Results and discussion

 The explanations of phytochemicals like polyphenols, tannins, saponins, it seems they don’t have activities as inhibitor for demethylase CYP51. Or these are different activities as anti fungi?

**Answer**:

**Thank you for your question and suggestion**.

 The author will try to answer this question. Not all of the secondary metabolites in the phytochemicals of P. crocatum have antifungal activity. Based on the literature, only secondary metabolites are included in a group of compounds such as phenols, polyphenols, tannins, saponins, and flavonoids that have antifungal activity. Antifungal activity has inhibitory mechanisms in several places such as inhibiting cell wall synthesis, destroying cell membrane permeability, inhibiting nucleic acid synthesis, and damaging fungal microtubules. The inhibition of lanosterol 14a demethylase in ergosterol biosynthesis will cause inhibition of ergosterol, a key enzyme that functions to maintain the integrity and function of cell membranes in fungal and main target to antifungal sites thereby impairing the permeability of the cell membrane. The antifungal activity contained in these compounds of P. crocatum will cause fungal cell lysis.

 9. Results and discussion

 “Based on the results of the review, it was found that P. crocatum contain compounds that have activity as an antifungal agent so it is expected to become a new antifungal agent that can slowly replace the extensive use of antibiotics. Therefore, new antifungal agents that safer, few side effects, cheaper, easier to get, and more potent against fungal infections are needed". I think these sentence need to be revised, since the sentences are unclear in particular the last sentence.

**Answer**:

**Thank you for your correction and suggestion**.

 The authors have revised this sentence.

 10. Conclusions

 The conclusion section need to be revised since there are grammatical error and unclear. For example: Natural products are an important resource…", should be “Natural products are important resources…”

**Answer**:

**Thank you for your correction and suggestion**.

 The authors have revised and followed the suggestions.

 11. Conclusions

 The conclusion should emphasize the antifungal activities of P. crocatum. Does P. crocatum have antifungal activities by inhibiting ergosterol synthesis or as a lanosterol 14α demethylase CYP51 inhibitor.

**Answer**:

**Thank you for your correction and suggestion**.

 The authors have revised and followed the suggestions.

# Peer review: Reviewer response for version 2

**DOI:** 10.5256/f1000research.145688.r167864

**Published:** 2023-04-12

**Authors:** Zubaida Yousaf

**Affiliations:** 1 Lahore College for Women University, Lahore, Pakistan

I would like to start by thanking the authors for addressing my previous comments and making the necessary changes to improve the quality of the manuscript.

 Overall, I believe that the authors have done an excellent job of revising the manuscript. They have carefully addressed all of my previous concerns and have provided additional data to support their claims.

 In conclusion, I would like to recommend that the revised manuscript be Approved for indexing.

Is the review written in accessible language?

Yes

Are all factual statements correct and adequately supported by citations?

Yes

Are the conclusions drawn appropriate in the context of the current research literature?

Yes

Is the topic of the review discussed comprehensively in the context of the current literature?

Partly

Reviewer Expertise:

Genetics and genomics of medicinal plants

I confirm that I have read this submission and believe that I have an appropriate level of expertise to confirm that it is of an acceptable scientific standard.

# Response

**Published:** 2023-04-18

**Authors:** Dikdik Kurnia

**Affiliations:** Universitas Padjadjaran, Indonesia

Thank you for the decision.

 We are pleased to get a lot of valuable knowledge from the revisions suggested by the reviewer.

 It is an honor for us to be reviewed by you.

 Thank you.

# Peer review: Reviewer response for version 1

**DOI:** 10.5256/f1000research.137976.r163050

**Published:** 2023-02-22

**Authors:** Zubaida Yousaf

**Affiliations:** 1 Lahore College for Women University, Lahore, Pakistan

I have received the following manuscript for review from F1000Research: "Phytochemical profiling of *Piper crocatum* and its antifungal activity as Lanosterol 14 alpha demethylase CYP51 inhibitor: a review". The following sections required revisions and modifications: Title, abstract, introduction, results and discussion and references.

**Title:**

 Please ensure that you title clearly specifies whether your study focuses on fungal enzyme inhibition assay, mechanism of action or another topic. Or change your title according to the results and discussion. Your abstract and introduction is somewhat related to your title but not according to the results and discussion.

**Abstract:**

 There is no information found on these lines of abstract in your results and discussion:

*"Its antifungal mechanisms in fungal cells occur due to ergosterol especially lanosterol 14 alpha demethylase CYP51 inhibition as a result of 5,6 desaturase (ERG3) downregulation. P. crocatum has an antifungal activity by its phytochemical profiling that act against fungi by inhibiting the fungal cytochrome P 450 pathway, make damaging cell membranes, fungal growth inhibition, morphological changes, and fungal cell lysis".*

**Introduction:** 

 Difference in statement of study idea in introduction and abstract. 

**Results and discussion:**

- In heading "Ethno-botany and ethno-pharmacology of *P. crocatum*". Add ethno botanical data related to plant like traditional uses and ethno-pharmacological aspects of plant.
- In heading "The phytochemical and chemical structures of *P. crocatum". *Sentence structure needs improvement. Don't repeat prepositions in sentence making.
- Give reasons of bio active compounds (leaf extract and essential oils) you have mentioned in table 1 and figure 2. Do these compounds have anti fungal activities? 
- In heading "Antifungal properties and structure" these highlighted lines *"Ergosterol is a key enzyme in fungal specific sterols, cytochrome P450 enzyme in fungi derived from S. cerevisiae, belonging to the CYP51 (lanosterol 14-α-demethylase) family, so with inhibition of biosynthesis in ergosterol, will damage cell membranes"*. I think these are the exact lines that have reflection of title statement. So, you should provide references of this activity from different published articles here in results and discussion and also in introduction and abstract.
- In figure 3: Spelling mistakes in caption.

**References:**

 Following amendments are required in reference list and citations:

- Check references according to journal format.
- Citations should be according to standard format.
- If more than 3 authors in references use *et al* after first author name. If 1 or 2 authors then write single or both authors’ name with year like (Mohamed 2006), (Mohammad and Jakupovie 2006), or (Mohamed *et al.* 2006).
- In case of two or more citations in the text, the citations must be arranged in chronological order.
- Arrange references alphabetically.
- Remove repetition in references.

Is the review written in accessible language?

Yes

Are all factual statements correct and adequately supported by citations?

Yes

Are the conclusions drawn appropriate in the context of the current research literature?

Yes

Is the topic of the review discussed comprehensively in the context of the current literature?

Partly

Reviewer Expertise:

Genetics and genomics of medicinal plants

I confirm that I have read this submission and believe that I have an appropriate level of expertise to confirm that it is of an acceptable scientific standard, however I have significant reservations, as outlined above.
